# Mangiferin Alleviates Mitochondrial ROS in Nucleus Pulposus Cells and Protects against Intervertebral Disc Degeneration via Suppression of NF-*κ*B Signaling Pathway

**DOI:** 10.1155/2021/6632786

**Published:** 2021-06-11

**Authors:** Haichao Yu, Guowei Hou, Jiankang Cao, Yanyu Yin, Yunpeng Zhao, Lei Cheng

**Affiliations:** ^1^Department of Orthopedic, Qilu Hospital, Cheeloo College of Medicine, Shandong University, Jinan, 250012 Shandong, China; ^2^Department of Orthopedic, PKU Care Luzhong Hospital, Zibo, 255499 Shandong, China; ^3^Department of Pain, Qilu Hospital, Cheeloo College of Medicine, Shandong University, Jinan, 250012 Shandong, China; ^4^Department of Orthopedic, Yucun Hospital of Xinwen Mining Group Co. Ltd., Taian, 271200 Shandong, China

## Abstract

Intervertebral disc degeneration (IVDD), one of the most common clinical diseases worldwide, causes disc herniation and sciatica. Recent studies have identified the involvement of mitochondrial dysfunction, inflammatory responses, and extracellular matrix degradation in IVDD. Mangiferin is known to protect against various diseases by inhibiting oxidative stress, suppressing inflammation reaction, and relieving mitochondrial dysfunction. Whether mangiferin can alleviate IVDD remains to be elucidated. In the present study, human nucleus pulposus cells (HNPCs) and mouse intervertebral discs were cultured and stimulated with TNF-*α*, with or without treatment of mangiferin. Moreover, we established a rat needle puncture model and injected mangiferin into the intervertebral discs to verify its protective effect on IVDD. Furthermore, the activity of the NF-*κ*B signaling pathway was tested in vitro. Our results indicated that mangiferin alleviated the inflammatory response and reversed the loss of major intervertebral disc components. Besides, mangiferin reduced reactive oxygen species production, ameliorated mitochondrial damage, and decreased the expression of apoptosis-related parameters in stimulation of TNF-*α*. In addition, mangiferin antagonized the activation of the NF-*κ*B signaling pathway induced by TNF-*α*. Collectively, mangiferin antagonized mitochondrial ROS in NP cells and protected against IVDD by suppressing the activation of the NF-*κ*B signaling pathway, which might provide a potential therapeutic instrument for IVDD.

## 1. Introduction

Approximately 84% of people are estimated to have low back pain (LBP) and 10% of LBP cases result in chronic disability [1, 2]. According to recent studies, LBP is closely associated with intervertebral disc degeneration (IVDD), which interferes the structure and function of the intervertebral disc (IVD). Recent studies have reported critical roles for reactive oxygen species (ROS), oxidative stress, and subsequent mitochondrial dysfunction in the development of IVDD [3–5]. ROS exacerbate various pathological processes involved in IVDD, such as activating the NF-*κ*B and MAPK signaling pathways, increasing inflammatory cytokine production, and promoting apoptosis and senescence in IVD cells [6–8]. Mitochondrial dysfunction is a main cause of ROS production, and mitochondria are also a predominant target of ROS. High levels of ROS lead to the gradual deterioration of mitochondrial function, which triggers excess catabolism in nucleus pulposus (NP) cells and increases inflammation in the microenvironment of the IVD [8].

Inflammation is reported to be a major contributor to IVDD [9]. TNF-*α* is a critical proinflammatory molecule involved in IVDD. TNF-*α* leads to a reduction in aggrecan and type II collagen (Col-2) levels and promotes the degradation of the extracellular matrix (ECM) by inducing the production of matrix metalloproteinases (MMPs) and a disintegrin and metalloproteinase with thrombospondin motifs (ADAMTSs) [10–13]. Moreover, TNF-*α* is known to promote ROS production and the release of various inflammatory cytokines, which potentially enhance cell apoptosis and senescence [14]. These pathological changes caused by TNF-*α* ultimately lead to the occurrence and development of IVDD.

Mangiferin (2-C-*β*-D-glucopyranosyl-1,3,6,7-tetrahydroxyxanthone), which has a C-glucosyl linkage with multiple -OH groups, possesses strong free radical-scavenging activity [15]. Mangiferin is extensively distributed in plants worldwide, but its principal and most accessible source is the mango tree. Mangiferin possesses a wide range of properties with pharmacological potential, including antioxidant, anti-inflammatory, antidiabetic, antihyperlipidemic, and antiatherogenic properties [16]. Mangiferin antagonizes oxidative stress and mitochondrial dysfunction in various pathological processes [17–19]. Mangiferin reduces artificially induced apoptosis and ECM degradation related to arthritis [20]. Additionally, mangiferin was shown to exert a protective effect on a rat spinal cord injury model by inhibiting the inflammatory response and apoptosis [21]. However, researchers have not clearly determined whether mangiferin plays a role in the development of IVDD. Building upon the results of previous studies, we will explore the role of mangiferin in preventing IVDD and then investigate the potential mechanisms involved.

## 2. Materials and Methods

### 2.1. Ethics Statement

In this study, human IVD tissues were obtained from 10 patients (4 males and 6 females; 18-30 years old) with IVDD (all with grade II IVDD) who underwent lumbar spine surgery at Qilu Hospital of Shandong University, Jinan, China. The degree of IVDD was assessed using the modified Pfirrmann grading system [22]. All patients involved in this study were given informed consent documents and voluntarily agreed to participate in this research, and this study was approved by the Medical Ethics Committee of Qilu Hospital of Shandong University. All animal experimental procedures were performed in accordance with the International Guiding Principles for Animal Research and were approved by the Laboratory Animal Centre of Shandong University.

### 2.2. Primary Human Nucleus Pulposus Cell (HNPC) Isolation

We collected the human IVD samples during spine surgeries. All procedures involved in the isolation and culturing of primary HNPCs were performed as previously reported [23]. We washed the blood from the IVD with precooled PBS. Then, we used a microscope to carefully remove the cartilage endplate and the annulus fibrosus and isolate the NP tissues, which were cut into fragments of approximately 2 mm. The NP fragments were digested with trypsin for 20 minutes and then with type II collagenase for 8 h and filtered through a 200-mesh sieve. The isolated NP cells were seeded in DMEM/F12 (HyClone, USA) containing 10% fetal bovine serum (FBS) (Gibco, USA), 50 mg/ml ascorbic acid, and 1% penicillin and streptomycin (HyClone, USA) at 37°C in an atmosphere containing 5% CO_2_ and 95% air at pH 7.2. The culture medium was replaced once every 3 days. All HNPCs were passaged when they reached approximately 80–90% confluence, and the indicated experiments were subsequently conducted.

### 2.3. HNPC Culture

HNPCs were seeded in a 6-well plate (1 × 10^6^ cells/ml). Then, the cells were treated with PBS, TNF-*α* (20 ng/ml) (PeproTech, USA), TNF − *α* (20 ng/ml) + mangiferin (100 *μ*M/ml) (MedChemExpress, China), and TNF − *α* (20 ng/ml) + mangiferin (500 *μ*M/ml).

### 2.4. Mouse IVD Isolation and Culture

All surgical procedures were performed under sterile conditions. The specimens of mouse spines were obtained from 10-week-old mice. After complete dissection of the spine and removal of the surrounding soft tissues, the lumbar spine was cut to a length of 5 mm while maintaining the integrity of the IVD. The lumbar spines of the mice were soaked in 100 U/ml penicillin/streptomycin for 30 minutes. The IVD tissues were incubated and stimulated with PBS (*n* = 7) or TNF-*α* (20 ng/ml, *n* = 7) in the presence or absence of mangiferin (100 *μ*M/ml, *n* = 7; 500 *μ*M/ml, *n* = 7) for 7 days [24]. All IVD tissues were collected for use in subsequent experiments.

### 2.5. Rats

Three-month-old Sprague-Dawley rats were purchased from the Animal Center of Shandong University. All animals were housed under controlled identical, standard specific pathogen-free (SPF) environmental conditions (23 ± 2°C, 12 h light/dark cycle) with free access to food and allowed to move freely. Local administration of mangiferin into the rat caudal disc via puncture was performed as previously reported [25]. Briefly, 2 *μ*l of PBS (*n* = 5) with or without 0.2 *μ*g of mangiferin (0 *μ*g, *n* = 5; 0.2 *μ*g, *n* = 5) was delivered into the punctured disc through an intradiscal injection via a 33-gauge Hamilton syringe (Hamilton Co., NV, USA) 3 days after puncture.

### 2.6. Real-Time PCR Analysis

HNPCs were cultured for 1 or 24 h using the methods described above to detect different indicators [23]. TRIzol reagent (Takara Bio, Japan) was used to extract total RNA from the HNPCs that had been cocultured with 20 ng/ml TNF-*α* and different concentrations of mangiferin. A cDNA synthesis kit (Toyobo, Japan) was used to reverse transcribe identical amounts of RNA into cDNAs according to the manufacturer's instructions. Real-time PCR was performed with SYBR Green PCR Master Mix (Toyobo, Japan) on a thermal cycler (Roche, Switzerland); the results were normalized to GAPDH. The specific PCR products for each gene were confirmed by performing a melting curve analysis. We used the mean from the control group as value 1. The relative gene expression levels were calculated using the formula 2^−*ΔΔ*Ct^. The sequence-specific primers used in the present study are listed in Table 1.

### 2.7. Western Blotting

HNPCs were cultured for 1 or 48 h using the methods described above to detect different indicators by Western blotting [23]. Proteins were extracted from HNPCs and mouse IVD tissues from each treatment group. Equal amounts of protein were separated on 10% and 12% acrylamide gels using SDS–PAGE and subsequently transferred to polyvinylidene difluoride (PVDF) membranes (Millipore, USA). After an incubation with 5% nonfat milk or 5% BSA for 1 h at room temperature, the membranes were incubated with the following primary antibodies (diluted 1 : 500–1 : 1000) at 4°C for 12 h: rabbit anti-iNOS (NB300-605 Novus, UK), rabbit anti-COX-2 (ab179800, Abcam), rabbit anti-ADAMTS-5 (ab41037, Abcam), rabbit anti-collagen-II (ab34712, Abcam), mouse anti-aggrecan (ab3778, Abcam), rabbit anti-Bcl-2 (AF6139, Affinity), rabbit anti-MMP-13 (AF5355, Affinity), rabbit anti-mitochondrial transcription factor A (TFAM) (AF0531, Affinity), rabbit anti-Optic Atrophy 1 (OPA1) (DF8587, Affinity), rabbit anti-Dynamin-related protein 1 (Drp1) (DF7037, Affinity), rabbit anti-cleaved-caspase-3 (c-caspase-3) (AF7022, Affinity), rabbit anti-Bax (#2772, Cell Signaling Technology), rabbit anti-I*κ*B*α* (#9242, Cell Signaling Technology), rabbit anti-phosphorylated I*κ*B*α* (pI*κ*B*α*) (#2859, Cell Signaling Technology), rabbit anti-p65 (#8242, Cell Signaling Technology), and rabbit anti-phosphorylated p65 (pp65) (#3033, Cell Signaling Technology). PVDF membranes were also incubated with a rabbit anti-GAPDH antibody (1 : 5000, Proteintech, USA) to ensure equal protein loading. After washes with Tris-buffered saline- (TBS-) Tween 20, the membranes were then incubated with the secondary antibody (1 : 5000, Cell Signaling Technology, USA) for 1 h, and the immunolabeled bands were detected using a Fluor Chem E Chemiluminescent Western Blot Imaging System (Amersham Imager 600, GE Amersham, USA). The data were analyzed using the ImageJ software (National Institutes of Health, USA).

### 2.8. X-Ray and Magnetic Resonance Imaging (MRI) Analyses

Seven days after the initial puncture, each group of rats was randomly selected to undergo X-ray and MRI scans before sacrifice [25]. The rats were maintained in a supine position with their tails straight for placement on a molybdenum target radiographic-image unit (GE, Boston, MA, USA). Radiographs were captured at a collimator-to-film distance of 66 cm, an exposure of 63 mAs, and a penetration power of 35 kV. We performed MRI using a 1.5 T system (GE) to obtain T2-weighted images (repetition time: 3000 ms; echo time: 80 ms; field of view: 200 mm^2^; slice thickness: 1.4 mm) in the coronal plane. All the radiographic images were saved in a Neusoft PACS/RIS DICOM 3.0 medical imaging system (Neusoft, Jinan, China). The IVD height and the adjacent upper and lower vertebral body heights were measured using Neusoft PACS/RIS measuring tools, and the disc height index (DHI) was calculated from these values.

### 2.9. Immunohistochemical (IHC) Staining and Hematoxylin and Eosin (HE) Staining

The IVD tissues collected from mice and rats in each treatment group were immersed in 4% paraformaldehyde for 72 h and then subjected to decalcification, after which they were embedded in paraffin and cut into 5 *μ*m sections. Trypsin (0.125%, Gibco, Waltham, USA) was used for antigen retrieval, and 3% hydrogen peroxide was used to eliminate endogenous peroxidase activity. All primary antibodies were diluted in 1× antibody dilution buffer (1× PBS with 10% v/v normal donkey serum (Solarbio, Beijing, China) and 0.01% Proclean (Beyotime Biotechnology, Shanghai, China)), and the serial sections were incubated with primary antibodies (rabbit anti-collagen-II, rabbit anti-MMP-13, and mouse anti-aggrecan; all 1 : 100, Abcam, UK) at 4°C for 12 h. Then, the sections were incubated with goat anti-rabbit and anti-mouse immunoglobulin- (IgG-) horseradish peroxidase (HRP) secondary antibodies (1 : 200, ZSGB-Bio, Beijing, China) for 60 minutes at room temperature. HE staining was performed according to the manufacturer's protocol. An IX71-SIF microscope (Olympus, Tokyo, Japan) was used to obtain the images.

### 2.10. Immunofluorescence (IF) Staining

HNPCs were seeded on cell coverslips and cultured with PBS, TNF-*α* (20 ng/ml), TNF − *α* (20 ng/ml) + mangiferin (100 *μ*M/ml), or TNF − *α* (20 ng/ml) + mangiferin (500 *μ*M/ml) for 48 h [26] before the levels of the iNOS, COX-2, MMP-13, and Col-2 proteins were detected. The cells were also cultured with PBS, TNF − *α* (20 ng/ml) + mangiferin (100 *μ*M/ml), or TNF − *α* (20 ng/ml) + mangiferin (500 *μ*M/ml) for 1 h, and the level of the NF-*κ*B pp65 protein was detected. The cells were washed 3 times with PBS, fixed with 4% paraformaldehyde for 20 minutes, permeabilized with 0.2% Triton X-100 for 15 minutes, and blocked with 1% BSA for 30 minutes at room temperature. Then, the cells were incubated with rabbit anti-iNOS (1 : 100, Novus, UK), rabbit anti-COX-2, rabbit anti-MMP-13, rabbit anti-collagen-II (all 1 : 100, Abcam, UK), rabbit anti-NF-*κ*B p65 (1 : 100, AF5006, Affinity, USA), and rabbit anti-NF-*κ*B pp65 (1 : 100, AF2006, Affinity, USA) primary antibodies for 12 h at 4°C. Afterwards, the cells were incubated with a fluorescently labeled goat anti-rabbit immunoglobulin (IgG) secondary antibody (1 : 50, ZSGB-Bio, Beijing, China) for 1 h at room temperature.

Trypsin (0.125%, Gibco, USA) was used for antigen repair of IVD tissues in mice, and 3% hydrogen peroxide was used to eliminate endogenous peroxidase activity. All primary antibodies were diluted in 1× antibody dilution buffer (1× PBS with 10% v/v normal donkey serum (Solarbio, Beijing, China) and 0.01% Proclean (Beyotime Biotechnology, Shanghai, China)), and the mouse IVD tissues were incubated with primary antibodies (rabbit anti-COX-2 and MMP-13, all 1 : 100, Abcam, UK) at 4°C for 12 h. Then, the tissues were incubated with a fluorescently labeled goat anti-rabbit IgG secondary antibody (1 : 50, ZSGB-Bio, Beijing, China) for 1 h at room temperature. The images were captured using a fluorescence microscope (Ti2-U, Nikon, Japan).

### 2.11. Safranin O and Fast Green Staining

Safranin O and fast green staining were performed to determine changes in proteoglycans. The mouse and rat IVD sections were stained with a Safranin O kit (Solarbio, China) according to the manufacturer's recommended procedure. An IX71-SIF microscope (Olympus, Tokyo, Japan) was used to capture the images.

### 2.12. TUNEL Assay

TUNEL staining of mouse IVD tissues from each treatment group cultured as described above for 7 days and HNPCs from each treatment group cultured as described above for 24 h [27] was performed using the TUNEL System (Elabscience, Wuhan, China) according to the manufacturer's protocol. The images were captured using a fluorescence microscope (Ti2-U, Nikon, Japan).

### 2.13. JC-1 Assay

HNPCs were cultured as described above for 24 h [27]. A JC-1 assay kit was used (Beyotime Biotechnology, China) to detect the mitochondrial membrane potential. In accordance with the manufacturer's instructions, primary HNPCs from each group cultured in 24-well plates were stained with the JC-1 staining solution at 37°C for 20 minutes. Then, the cells in each well were washed twice with 1× JC-1 staining buffer. The images were captured using a fluorescence microscope (Ti2-U, Nikon, Japan).

### 2.14. ROS Assay

HNPCs were cultured as described above for 24 h [27]. We used a ROS assay kit (Beyotime Biotechnology, China) to detect intracellular ROS levels. All procedures were performed according to the manufacturer's instructions. Briefly, after two washes with sterile PBS, HNPCs were stained with 10 *μ*M DCFDA at 37°C for 20 minutes in the dark and mixed every 15 minutes. Then, the HNPCs were washed three times with serum-free culture medium to reduce interference from excess DCFDA. The images were captured using a fluorescence microscope (Ti2-U, Nikon, Japan).

### 2.15. MitoTracker Assay

HNPCs were cultured according to the above method for 24 h [27], and MitoTracker staining was performed to visualize the mitochondria in the HNPCs of each experimental group. The procedure was performed in accordance with the instructions of the MitoTracker assay kit (MitoTracker Red CMXRos, Beyotime Biotechnology, China). HNPC nuclei were stained with DAPI, while the cytoskeleton was stained with phalloidin (green) (Solarbio, China). The images were captured using a fluorescence microscope (Ti2-U, Nikon, Japan).

### 2.16. Transmission Electron Microscopy (TEM)

HNPCs were dehydrated in a graded series of ethanol solutions (50%, 70%, 80%, 90%, 95%, 100%, and 100%) for 15 minutes per solution and infiltrated with propylene oxide embedding medium overnight. Ultrathin sections (50 nm) were obtained using an EMUC7 ultramicrotome (Leica), poststained with uranyl acetate and lead citrate and visualized using a transmission electron microscope (HT7700; Hitachi, Japan).

### 2.17. Statistical Analysis

All data are presented as the mean values ± standard deviations (SD) and were analyzed using the GraphPad Prism v.5.0 software (GraphPad Software Inc., USA). Comparisons of various treatment groups were performed using analysis of variance (ANOVA) with Tukey's post hoc test. *P* < 0.05 was considered statistically significant.

## 3. Results

### 3.1. Mangiferin Alleviated the TNF-*α*-Induced Inflammatory Response in HNPCs

**Figure 1:**
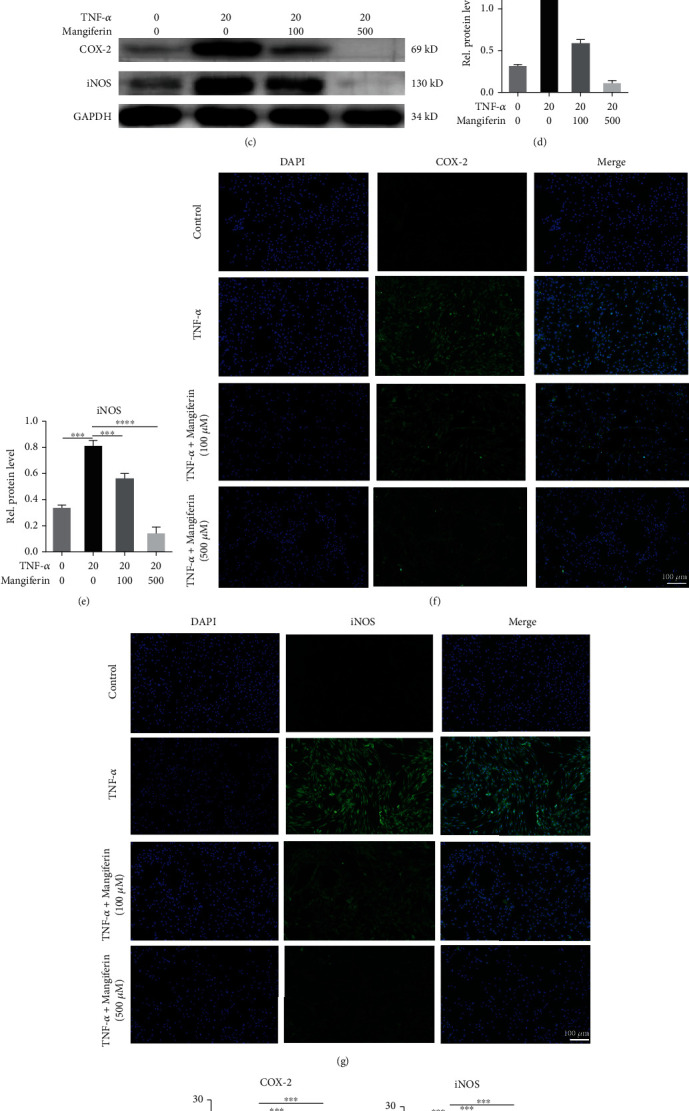
Mangiferin alleviated the inflammatory response in HNPCs. HNPCs were stimulated with PBS, TNF-*α* (20 ng/ml), TNF − *α* (20 ng/ml) + mangiferin (100 *μ*M/ml), or TNF − *α* (20 ng/ml) + mangiferin (500 *μ*M/ml). Total mRNA was extracted from each group, and the expression levels of (a) COX-2 and (b) iNOS were assayed using real-time PCR. Total protein was extracted from each group, and (c–e) the expression of COX-2 and iNOS was evaluated using Western blotting. (f–i) The expression levels of COX-2 and iNOS were detected using IF staining. Scale bar: 100 *μ*m. All the experiments were repeated at least three times. Significant differences are indicated as follows: ^∗^*P* < 0.05, ^∗∗^*P* < 0.01, ^∗∗∗^*P* < 0.001, and ^∗∗∗∗^*P* < 0.0001.

Previous research has shown that increased expression of inflammatory cytokines is an important factor leading to IVDD. Therefore, we investigated the role of mangiferin in the progression of IVDD by culturing HNPCs with PBS, TNF-*α* (20 ng/ml), TNF − *α* (20 ng/ml) + mangiferin (100 *μ*M/ml), or TNF − *α* (20 ng/ml) + mangiferin (500 *μ*M/ml) for 24 h to detect the mRNA level and 48 h to detect the protein level. After treatment, mRNA was extracted from the HNPCs, followed by real-time PCR. As indicated in Figures 1(a) and 1(b), after TNF-*α* stimulation, the expression levels of COX-2 and iNOS were increased by 2.74 and 29.31 times compared with the PBS group. Coculture with different concentrations of mangiferin (100 *μ*M/ml and 500 *μ*M/ml), the expression of these cytokines decreased by 82.16% and 83.75% and 97.19% and 97.52%, respectively, compared with the TNF-*α* group. Next, total protein was extracted from treated HNPCs, and Western blotting was performed to detect the expression levels of COX-2 and iNOS. As shown in Figures 1(c)–1(e), the lowest expression levels of these inflammatory cytokines were detected in the PBS group and the highest levels were detected in the TNF-*α* group, and the protein levels of COX-2 and iNOS in the TNF-*α* group were 3.91 and 2.62 times higher than those in the PBS group. After treatment with different concentrations of mangiferin, the expression levels of these inflammatory cytokines decreased in a dose-dependent manner, the levels of COX-2 and iNOS were reduced by 51.58% and 90.94% and 35.74% and 84.75%, respectively. As shown in Figures 1(f)–1(i), as measured using IF staining, in the four groups, the mean fluorescence intensities of COX-2 and iNOS were 1.94, 22.08, 9.38, and 7.57 and 1.91, 25.92, 11.72, and 5.65, respectively. The statistical analysis of the mean fluorescence intensities indicated that the expression levels of COX-2 and iNOS in the TNF-*α* group were 11.38 and 13.57 times higher than those in the PBS group, respectively. The mean fluorescence intensities of COX-2 and iNOS in the mangiferin (100 *μ*M/ml) and mangiferin (500 *μ*M/ml) groups decreased by 57.52% and 65.72% and 54.78% and 78.20%, respectively, compared with those in the TNF-*α* group. Therefore, mangiferin reduced the TNF-*α*-induced increases in the expression levels of COX-2 and iNOS, which alleviates the inflammatory response during the process of IVDD.

### 3.2. Mangiferin Restrained ECM Metabolism and Alleviated the Degeneration of HNPCs

**Figure 2:**
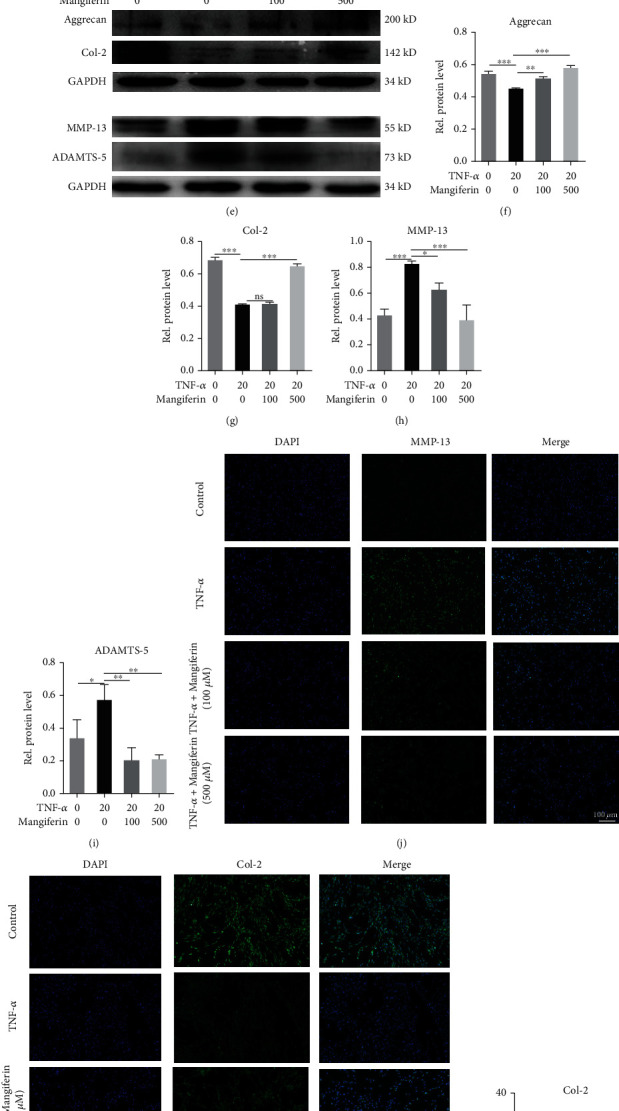
Mangiferin inhibited the TNF-*α*-induced increase in the expression of catabolic enzymes and alleviated the loss of major ECM components. HNPCs were treated with PBS, TNF-*α* (20 ng/ml), TNF − *α* (20 ng/ml) + mangiferin (100 *μ*M/ml), or TNF − *α* (20 ng/ml) + mangiferin (500 *μ*M/ml). The expression levels of (a) aggrecan, (b) Col-2, (c) MMP-13, and (d) ADAMTS-5 were measured using real-time PCR. (e–i) Levels of the aggrecan, Col-2, MMP-13, and ADAMTS-5 protein were assayed using Western blotting. (j–m) The expression levels of MMP-13 and Col-2 were detected using IF staining. Scale bar: 100 *μ*m. All the experiments were repeated at least three times. Significant differences are indicated as follows: ns *P* > 0.05, ^∗^*P* < 0.05, ^∗∗^*P* < 0.01, ^∗∗∗^*P* < 0.001, and ^∗∗∗∗^*P* < 0.0001.

As TNF-*α* promotes the expression of ADAMTSs and MMPs, leading to changes in the synthesis of ECM molecules and increased ECM degradation, the increased expression of MMP-13 and ADAMTS-5 in degenerative IVD tissues results in decreases in Col-2 and aggrecan levels [28, 29]. Therefore, we established an HNPC inflammation model to mimic the pathological process and verified the role of mangiferin in IVDD. In this study, HNPCs were cultured and stimulated with PBS, TNF-*α* (20 ng/ml), TNF − *α* (20 ng/ml) + mangiferin (100 *μ*M/ml), or TNF − *α* (20 ng/ml) + mangiferin (500 *μ*M/ml) 24 h to detect the mRNA level and 48 h to detect the protein level. As shown in Figures 2(a) and 2(b), the levels of the aggrecan and Col-2 mRNAs decreased by 67.18% and 88.91% in TNF-*α* group than those in PBS group, while the production of Col-2 and aggrecan gradually increased by 1.48 and 1.87 and 1.51 and 3.47 times after treatment with different concentrations of mangiferin. As shown in Figures 2(c) and 2(d), the mRNA levels of the MMP-13 and ADAMTS-5 after TNF-*α* stimulation were 4.07 and 4.25 times higher than those in PBS group, respectively. Compared with the TNF-*α* group, the expression levels of these cytokines gradually decreased by 63.94% and 89.44% and 39.64% and 64.83% after treatment with 100 *μ*M/ml and 500 *μ*M/ml mangiferin. We extracted total proteins from HNPCs after cultivation with TNF-*α* and mangiferin to further explore the role of mangiferin in inhibiting the degeneration of HNPCs. As shown in Figures 2(e)–2(i), higher expression levels of MMP-13 and ADAMTS-5 were detected in TNF-*α*-stimulated HNPCs than in HNPCs treated with PBS alone, while lower expression levels of aggrecan and Col-2 were observed in TNF-*α*-stimulated HNPCs than in PBS-treated HNPCs. The expression levels of MMP-13 and ADAMTS-5 were significantly decreased in response to the mangiferin treatment compared with those in the TNF-*α* group, while the expression levels of aggrecan gradually increased. Compared with TNF-*α* group, the expression of Col-2 was not increased after 100 *μ*M/ml of mangiferin treatment, while the expression of Col-2 was increased after 500 *μ*M/ml of mangiferin treatment. These results were verified by IF staining, as shown in Figures 2(j)–2(m), in the four groups, and the statistical analysis of the mean fluorescence intensities indicated that the expression levels of MMP-13 in the TNF-*α* group were 2.73 times higher than those in the PBS group, and the expression levels of Col-2 in the TNF-*α* group decreased by 63% than those in the PBS group. The mean fluorescence intensities of MMP-13 in the mangiferin (100 *μ*M/ml) and mangiferin (500 *μ*M/ml) groups decreased by 59.19% and 60.10% compared with those in the TNF-*α* group, and the expression levels of Col-2 in the mangiferin (100 *μ*M/ml) and mangiferin (500 *μ*M/ml) groups were 1.02 and 2.05 times higher than those in the TNF-*α* group. Taken together, the experimental results shown in Figures 1 and 2 indicate that mangiferin may reduce the inflammatory response and the production of matrix-degrading enzymes in HNPCs and alleviate the loss of major IVD components that occurs during the development of IVDD.

### 3.3. Mangiferin Alleviated TNF-*α*-Induced Oxidative Stress and Mitochondrial Dysfunction in HNPCs

**Figure 3:**
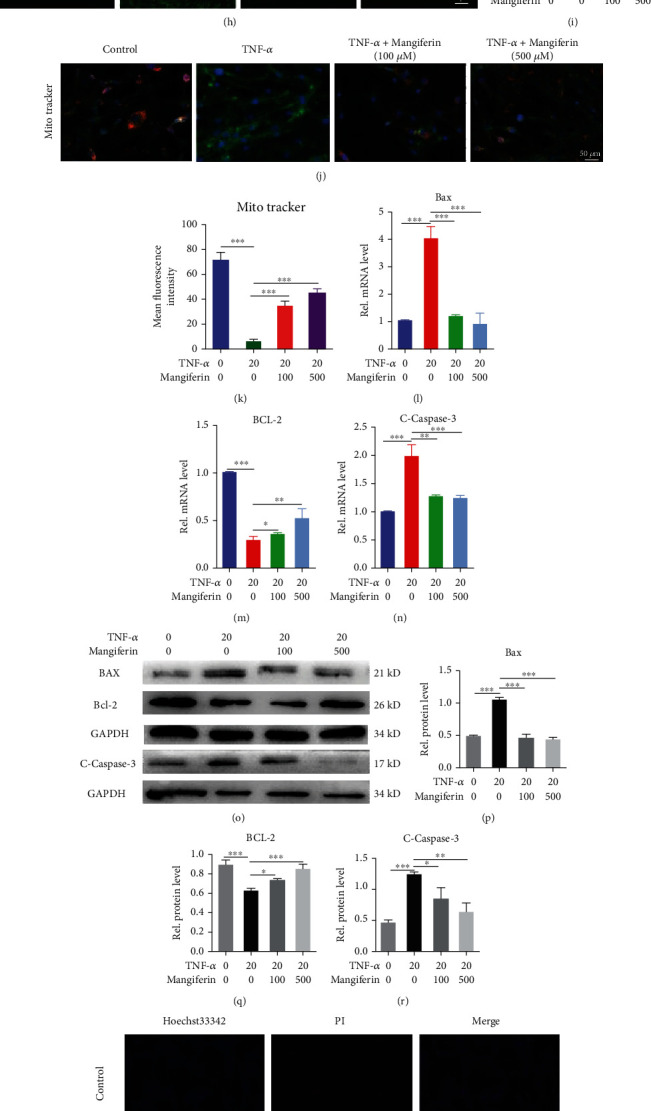
Mangiferin alleviated TNF-*α*-induced mitochondrial dysfunction, oxidative stress, and apoptosis in HNPCs. HNPCs were treated with TNF-*α* (20 ng/ml) and different concentrations of mangiferin (100 *μ*M/ml and 500 *μ*M/ml). (a) The mitochondrial morphology in HNPCs was observed using TEM. Scale bars: 5 *μ*m and 1 *μ*m. (b–e) The levels of OPA1, TFAM, and Drp1 were assayed using Western blotting. (f, g) JC-1 was used to detect the mitochondrial membrane potential in HNPCs. Scale bar: 100 *μ*m. (h, i) ROS levels were detected in HNPCs from each treatment group. Scale bar: 100 *μ*m. (j, k) MitoTracker was used to detect the mitochondrial membrane potential of HNPCs. Scale bar: 25 *μ*m. The expression of (l) Bax, (m) Bcl-2, and (n) c-caspase-3 in each indicated group was assayed using real-time PCR. (o–r) The levels of Bax, Bcl-2, and c-caspase-3 were assayed using Western blotting. (s, u, v) Hoechst 33342 and (t, w) TUNEL staining of the HNPCs in each indicated group. Scale bar: 100 *μ*m. All the experiments were repeated at least three times. Significant differences are indicated as follows: ns *P* > 0.05, ^∗^*P* < 0.05, ^∗∗^*P* < 0.01, ^∗∗∗^*P* < 0.001, and ^∗∗∗∗^*P* < 0.0001.

We cultured HNPCs with PBS, TNF-*α* (20 ng/ml), TNF − *α* (20 ng/ml) + mangiferin (100 *μ*M/ml), or TNF − *α* (20 ng/ml) + mangiferin (500 *μ*M/ml) to verify whether mangiferin inhibits mitochondrial dysfunction caused by TNF-*α*-induced oxidative stress in HNPCs. We used TEM to observe the mitochondrial morphology of HNPCs. As shown in Figure 3(a), the mitochondria were swollen and deformed and exhibited disrupted mitochondrial cristae after HNPCs were treated with TNF-*α*. However, treatment with different concentrations of mangiferin gradually reversed this phenomenon, which may prove that mangiferin plays a role in protecting against mitochondrial damage in HNPCs. OPA1, Drp1, and TFAM are biomarkers of mitochondrial morphology and function [30, 31], as shown in Figures 3(b)–3(e), and our study found that the mitochondrial dysfunction caused by TNF-*α* can be alleviated after treatment with mangiferin. As shown in Figures 3(f), 3(g), 3(j), and 3(k), JC-1 and MitoTracker staining were performed to detect the mitochondrial membrane potential, and our results further proved that mangiferin reversed the TNF-*α*-induced mitochondrial damage in HNPCs. According to previous studies, ROS production increases in degenerative IVD tissue and forms a positive feedback loop that further promotes the production of ROS. In addition, mitochondria are the main target of ROS attack, and inhibiting the production of ROS may become an important part of IVDD treatment. As shown in Figures 3(h) and 3(i), the statistical analysis of the DCFDA indicated that the expression levels of ROS in the TNF-*α* group were 11.38 times higher than those in the PBS group. The DCFDA indicated that the expression levels of ROS in the mangiferin (100 *μ*M/ml) and mangiferin (500 *μ*M/ml) groups decreased by 80.52% and 91.66%, respectively, compared with those in the TNF-*α* group.

HNPC apoptosis has been shown to play a pivotal role in the degeneration of the IVD, over the past few years, and apoptosis in the IVD has become a focus area of research [8]. Relevant studies have mainly investigated three apoptotic signaling pathways: the mitochondrial pathway, the death receptor pathway, and the endoplasmic reticulum pathway. Among these pathways, the mitochondrial pathway plays an important role in the apoptosis of IVD cells [32, 33]. After treating HNPCs using the method described above, we extracted the total RNA and proteins to examine markers of the mitochondrial apoptosis pathway. As illustrated in Figures 3(l)–3(r), compared with TNF-*α* group, after treatment with mangiferin at different concentrations, the mRNA expression levels of Bax and c-caspase-3 decreased by 72.30% and 78.53% and 82.16% and 83.75%, respectively, and increased the expression of Bcl-2 by 1.25 and 1.82 times, respectively. Consistent with the trend of mRNA, mangiferin can significantly decrease the protein levels of Bax and c-caspase-3, increase the protein level of Bcl-2, and play a protective role in the inhibition of apoptosis. As shown in Figures 3(s)–3(w), TNF-*α* increased the number of TUNEL-positive HNPCs compared with the number detected in HNPCs treated with PBS alone, but different concentrations of mangiferin prevented the TNF-*α*-induced increase in the number of TUNEL-positive HNPCs; furthermore, Hoechst 33342 staining also proved that mangiferin inhibited the apoptosis of HNPCs caused by TNF-*α*.

### 3.4. Mangiferin Protected against TNF-*α*-Induced Degeneration in Cultured Mouse IVD Tissues

**Figure 4:**
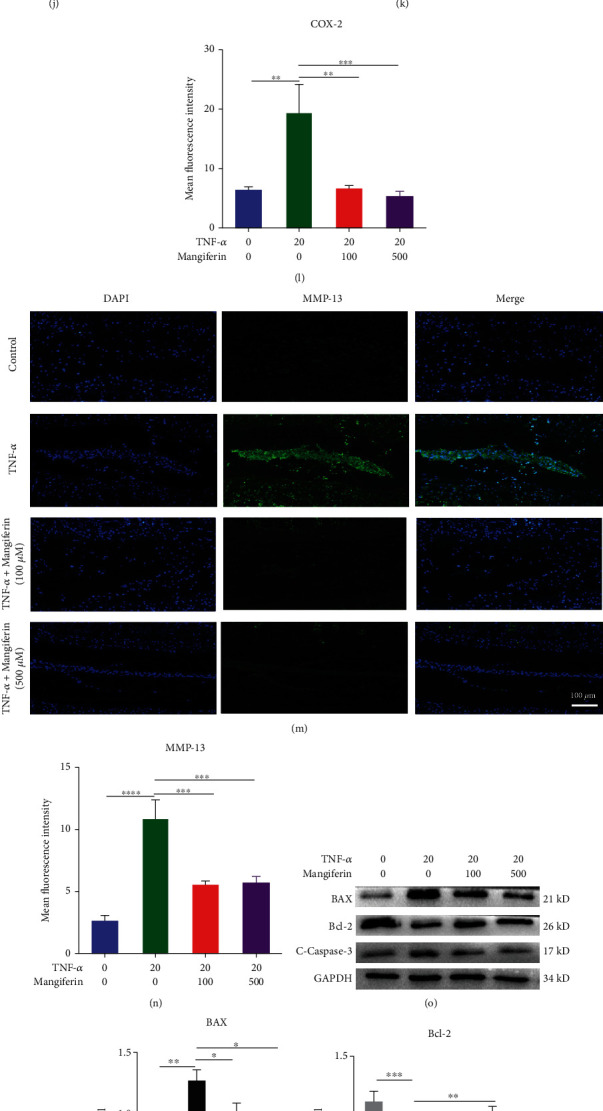
Mangiferin alleviated the pathological reactions associated with IVDD caused by TNF-*α* in cultured mouse IVD tissues. The IVD tissues from mice were treated with PBS (*n* = 7) or TNF-*α* (20 ng/ml, *n* = 7) in the presence or absence of mangiferin (100 *μ*M/ml, *n* = 7; 500 *μ*M/ml, *n* = 7). (a–e) Total protein was extracted, and levels of the COX-2, iNOS, ADAMTS-5, and MMP-13 proteins were evaluated using Western blotting. (f) Safranin O staining was performed after treating the mouse IVD tissues with TNF-*α* and different concentrations of mangiferin. Scale bar: 100 *μ*m. (g, i) The expression level of aggrecan in mouse IVD tissues was detected using IHC staining. Scale bar: 100 *μ*m. (h, j) IHC staining showing the level of Col-2 in mouse IVD tissues. Scale bar: 100 *μ*m. (k–n) We detected the expression levels of COX-2 and MMP-13 using IF staining. Scale bar: 100 *μ*m. (o–r) We detected the expression levels of Bax, Bcl-2, and c-caspase-3 using Western blotting. (s, t) TUNEL staining of the IVD tissues from mice in each indicated group. Scale bar: 100 *μ*m. All the experiments were repeated at least three times. Significant differences are indicated as follows: ^∗^*P* < 0.05, ^∗∗^*P* < 0.01, ^∗∗∗^*P* < 0.001, and ^∗∗∗∗^*P* < 0.0001.

We cultured mouse IVDs from the upper and lower vertebral bodies in vitro with PBS, TNF-*α* (20 ng/ml), TNF − *α* (20 ng/ml) + mangiferin (100 *μ*M/ml), or TNF − *α* (20 ng/ml) + mangiferin (500 *μ*M/ml) for 7 days. Total protein was extracted from the cultured mouse IVD tissues for Western blot analysis, as shown in Figures 4(a)–4(e). Compared with the PBS group, the expression levels of COX-2, iNOS, ADAMTS-5, and MMP-13 were increased by 3.06, 1.81, 1.91, and 2.01 times in the TNF-*α* group. After treatment with different concentrations of mangiferin, the expression of COX-2, iNOS, ADAMTS-5, and MMP-13 decreased by 28.22%, 18.16%, 24.84%, and 18.49% and 42.47%, 46.49%, 33.16%, and 29.61, respectively. As shown in Figures 4(g)–4(j), the expression of aggrecan and Col-2 was decreased in TNF-*α*-stimulated IVDs compared with IVDs treated with PBS alone. After treatment with two different concentrations of mangiferin, the levels of these inflammatory cytokines decreased in a dose-dependent pattern, and mangiferin mitigated the TNF-*α*-induced loss of aggrecan and Col-2. Safranin O staining is a method used to detect proteoglycans and glycosaminoglycans. We used Safranin O staining to evaluate the degree of IVDD in mice and verify the role of mangiferin in inhibiting IVDD. As shown in Figure 4(f), proteoglycans were decreased in the TNF-*α* group compared with the control group. After treatment with different concentrations of mangiferin, the proteoglycans in the IVD group were gradually increased compared with the TNF-*α* group. In Figures 4(k)–4(n), we used IF staining to determine the expression of COX-2 and MMP-13 in mouse IVD tissues and found that, compared with the PBS group, the expression of COX-2 and MMP-13 increased by 4.21 and 3.14 times in the TNF-*α* group. Compared with TNF-*α* group, the expression of MMP-13 and COX-2 decreased by 49.46% and 48.04% and 66.68% and 74.61%, respectively, after treatment with different concentrations of mangiferin. As illustrated in Figures 4(o)–4(r), we detected the expression of apoptotic proteins using Western blotting. Compared with the PBS group, the protein levels of the Bax and c-caspase-3 were increased by 1.67 and 1.38 times and the protein level of Bcl-2 was decreased by 49.48% in the TNF-*α* group. After treatment with 100 *μ*M/ml and 500 *μ*M/ml mangiferin, the level of Bcl-2 was increased by 1.41 and 1.74 times, and the levels of Bax and c-caspase-3 were decreased by 25.33% and 33.63% and 22.92% and 27.80, respectively. As illustrated in Figures 4(s) and 4(t), TUNEL-positive cells were detected in mouse IVD tissues to further verify the role of mangiferin in inhibiting HNPC apoptosis. Compared with that in the PBS group, a greater number of TUNEL-positive cells were observed in the TNF-*α* group, and the number of TUNEL-positive cells gradually decreased in response to the mangiferin treatment. Based on these results, mangiferin might protect against IVDD by reducing the apoptosis of HNPCs.

### 3.5. Mangiferin Alleviated the Degeneration of IVD in Rats In Vivo

**Figure 5:**
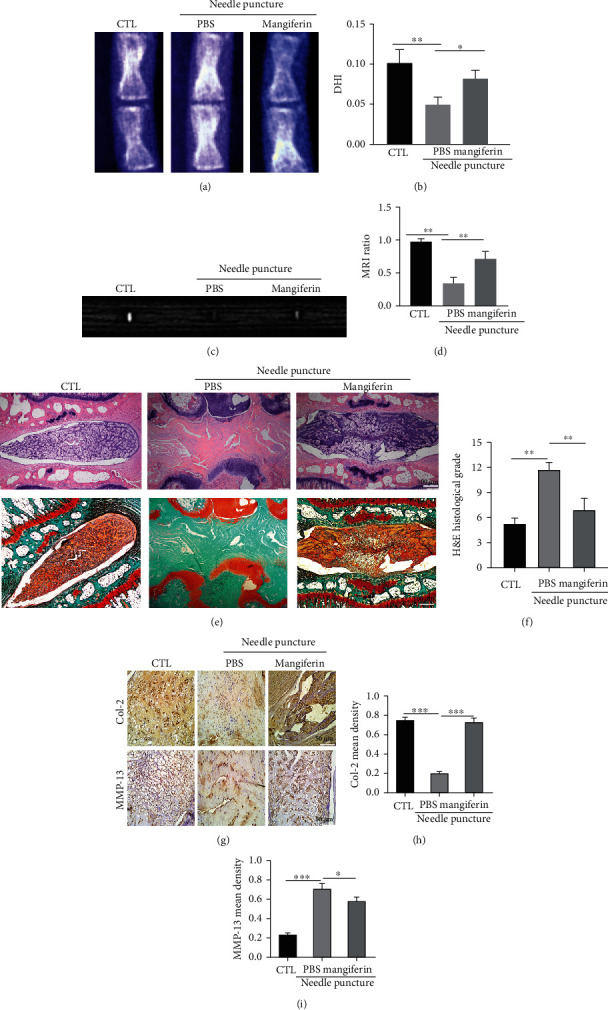
Mangiferin alleviated the degeneration of the rat IVD in vivo. (a, b) X-ray was obtained to measure the height of the intervertebral space in rats from each group (*n* = 5). (c, d) MRI was used to assess the degree of IVDD in rats from each group (*n* = 5). (e, f) HE and SO staining were performed to analyze the IVDs in rats from each group (*n* = 5). Scale bar: 100 *μ*m. (g–i) IHC was used to measure the expression of Col-2 and MMP-13 in each group (*n* = 5). All the experiments were repeated at least three times. Scale bar: 50 *μ*m. Significant differences are indicated as follows: ^∗^*P* < 0.05, ^∗∗^*P* < 0.01, ^∗∗∗^*P* < 0.001, and ^∗∗∗∗^*P* < 0.0001.

We established a rat IVD needle puncture model and injected mangiferin into the rat IVD to verify that mangiferin inhibits IVDD in rats. As shown in Figures 5(a) and 5(b), the IVD height was diminished after needle puncture, while mangiferin treatment largely attenuated this degeneration change of IVD, as assayed using X-ray measurements. As shown in Figures 5(c) and 5(d), MRI-T2WI detected a higher signal intensity in the rat IVD after mangiferin treatment than in the IVD of the needle puncture group, which proved that mangiferin plays an active role in the process of IVDD. As illustrated in Figures 5(e) and 5(f), HE staining revealed that mangiferin alleviated the decrease in the height of the intervertebral space associated with IVDD and improved the IVD morphology compared with the needle puncture group. Safranin O staining showed that mangiferin reduced the loss of proteoglycans during the process of IVDD. As shown in Figures 5(g)–5(i), the IHC results showed that injection of mangiferin alleviated the loss of Col-2 and reduced the production of MMP-13 associated with IVDD, which further validated the protective effect of mangiferin on IVDD.

### 3.6. Mangiferin Antagonized the Activation of the NF-*κ*B Signaling Pathway

**Figure 6:**
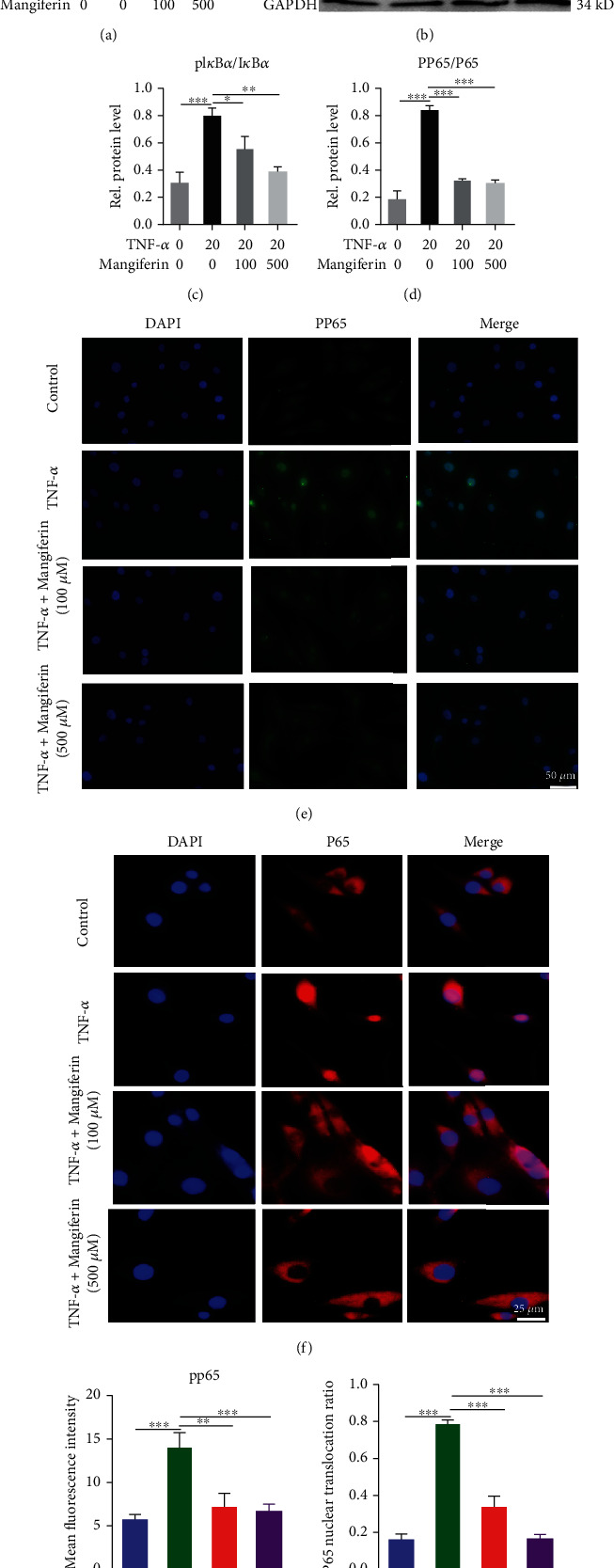
Mangiferin antagonized the TNF-*α*-induced activation of the NF-*κ*B signaling pathway. HNPCs were treated with PBS, TNF-*α* (20 ng/ml), TNF − *α* (20 ng/ml) + mangiferin (100 *μ*M/ml), or TNF − *α* (20 ng/ml) + mangiferin (500 *μ*M/ml). (a) Total mRNA was extracted, and the expression of NF-*κ*B_1_ was assayed using real-time PCR. (b–d) Total protein was extracted, and the levels of pI*κ*B*α* and pp65 were evaluated using Western blotting. (e, g) Representative images of IF staining for pp65 in cultured HNPCs from each group. Scale bar: 50 *μ*m. (f, h) Nuclear translocation of NF-*κ*B p65 in HNPCs as determined through IF staining. Scale bar: 25 *μ*m. All the experiments were repeated at least three times. Significant differences are indicated as follows: ^∗^*P* < 0.05, ^∗∗^*P* < 0.01, ^∗∗∗^*P* < 0.001, and ^∗∗∗∗^*P* < 0.0001.

Activation of the NF-*κ*B signaling pathway is known to play an important role in the development of IVDD. We stimulated HNPCs with PBS, TNF-*α* (20 ng/ml), TNF − *α* (20 ng/ml) + mangiferin (100 *μ*M/ml), and TNF − *α* (20 ng/ml) + mangiferin (500 *μ*M/ml) for 1 h and extracted total mRNA and total protein for analysis to explore whether mangiferin protects against IVDD by inhibiting the NF-*κ*B signaling pathway. Notably, pI*κ*B*α* and p65 are important parameters used to measure the activation of the NF-*κ*B signaling pathway [34]. As shown in Figure 6(a), compared with the PBS group, the expression level of NF-*κ*B_1_ increased 1.70 times after stimulation with TNF-*α* for 1 h, whereas the expression level of NF-*κ*B_1_ decreased by 42.15% and 51.12% after treatment with different concentrations of mangiferin. As shown in Figures 6(b)–6(d), compared with the PBS group, the protein levels of the pI*κ*B*α*/I*κ*B*α* and pp65/P65 increased after treatment with TNF-*α*, whereas their levels were decreased after treatment with different concentrations of mangiferin. In addition, the IF staining shown in Figures 6(e) and 6(g) indicated that the TNF-*α*-induced increase in the level of pp65 was abolished by treatment with mangiferin. As shown in Figures 6(f) and 6(h), HNPCs were collected and IF staining showed that different concentrations of mangiferin could eliminate TNF-*α*-induced p65 nuclear translocation.

## 4. Discussion

Previous studies have confirmed that IVDD is mediated by the abnormal production of proinflammatory molecules secreted by various cell types [35–37]; various proinflammatory cytokines and chemokines are reported to be associated with IVDD [38–41], among which TNF-*α* has been widely studied and shown to facilitate the development of IVDD. As mentioned above, TNF-*α* promotes the production of various inflammatory cytokines and causes ECM degradation, leading to increased catabolism of the IVD [42]; therefore, it is a promising target for the treatment of clinical IVDD. In our study, we established a TNF-*α*-induced inflammatory response model of HNPCs to explore the role of mangiferin in inhibiting IVDD and to investigate the mechanism of this effect. The expression levels of COX-2 and iNOS secreted by HNPCs were significantly elevated after treatment with TNF-*α* compared with those in the PBS group; consistent with previous studies, it was demonstrated that TNF-*α* caused the inflammatory response of HNPCs and resulted in the production of important inflammatory cytokines that contribute to IVDD. Intriguingly, after treatment with mangiferin, the expression of these inflammatory cytokines decreased compared with the TNF-*α* group. Furthermore, TNF-*α* increased the expression of MMPs and ADAMTSs, which leads to the destruction of the ECM, an important aspect of the pathological process of IVDD [29, 43]. In our study, TNF-*α* markedly increased the expression of MMP-13 and ADAMTS-5, leading to a significant decrease in aggrecan and Col-2 expression, which are the main components of the IVD, implying a certain level of anabolism [40]. Previous studies have shown that mangiferin alleviates Col-2 and aggrecan loss in tert-butyl hydroperoxide-stimulated chondrocytes [20]. In our vivo and vitro experiments, TNF-*α* enhanced the expression level of the catabolic markers MMP-13 and ADAMTS-5 and diminished the expression levels of the anabolic markers aggrecan and Col-2 compared with the control group. The addition of mangiferin at different concentrations alleviated the loss of Col-2 and aggrecan induced by TNF-*α* and decreased the expression of MMP-13 and ADAMTS-5, indicating that mangiferin might protect against disorganization of anabolism in IVDD.

Previous studies reported the abnormal morphology and dysfunction of mitochondria in aging NP cells [27], and mitochondrial dysfunction plays an important role in promoting the development of IVDD. In our study, compared with the control group, the mitochondria of HNPCs were swollen and deformed after TNF-*α* treatment, and the expression level of ROS was increased, which in turn led to inflammatory response of HNPCs and the damage of mitochondrial function, and mangiferin reduced these changes in mitochondria and ROS production. Moreover, mangiferin plays a protective role in mitochondrial function by affecting the expression of mitochondrial morphology and function-related indicators, thereby mitigating the process of IVDD by reducing mitochondrial dysfunction and ROS production. Apoptosis causes a significant decrease in the number of living and functional IVD cells, which results in increased expression of proinflammatory cytokines and exacerbates IVDD [8]. A large number of studies have shown that TNF-*α* induces the apoptosis of NP cells, which significantly increases the apoptotic rate and senescence [14, 44, 45]. The imbalance of Bax and Bcl-2 expression is involved in the mitochondrial apoptosis pathway during IVD degeneration, inducing the expression of c-caspase-3 and promoting the apoptosis of NP cells [46]. In our study, after TNF-*α* stimulation of HNPCs, we observed increased levels of Bax and c-caspase-3 and decreased levels of Bcl-2, and these results suggest that TNF-*α* could increase the expression of proapoptotic parameters and reduce the expression of antiapoptotic parameters and may be involved in mitochondrial apoptosis pathway. After treatment with mangiferin, the levels of Bax and c-caspase-3 decreased, and the expression of Bcl-2 increased. These results imply that mangiferin reduces the TNF-*α*-induced increase in the apoptosis rate of HNPCs, thus plays a role in relieving IVDD. To further validate our study, we further observed the effect of mangiferin on TNF-*α*-induced apoptosis of HNPCs by TUNEL and Hoechst 33342 staining. Compared with TNF-*α* stimulation group, mangiferin can alleviate the expression of inflammatory cytokines, reduce the loss of Col-2 and aggrecan, and alleviate the apoptosis of IVD in mice. Moreover, in vitro experiments further suggested that mangiferin can alleviate multiple pathological processes in IVDD.

In the in vivo experiment using rats, compared with the needle puncture group, intradiscal injection of mangiferin can alleviate the loss of IVD height and IVD major contents and reduce the expression of inflammatory cytokines. Therefore, the results suggest that mangiferin might play a protective role in IVDD in vivo and in vitro.

In addition, previous studies have reported that activation of the NF-*κ*B signaling pathway is involved in IVDD, activation of NF-*κ*B signaling pathway can accelerate IVDD, while antagonism of NF-*κ*B signaling pathway can alleviate the aging process [24]. TNF-*α* is involved in various pathological processes related to IVDD, mainly by activating the NF-*κ*B signaling pathway [26]. The detection of mRNA expression showed that mangiferin inhibited the increase in NF-*κ*B_1_ expression caused by TNF-*α*. Levels of the pI*κ*B and pp65 proteins, which are critical parameters for investigating the activity of the NF-*κ*B signaling pathway [34], also increased after treatment with TNF-*α*. The levels of pI*κ*B and pp65 were significantly reduced by mangiferin treatment. In addition, nuclear translocation of p65 determines the activity of NF-*κ*B signaling pathway [47]. Moreover, after treatment with mangiferin, nuclear translocation of p65 can be inhibited, so it plays an important and positive role in blocking the activation of NF-*κ*B signaling pathway.

In conclusion, mangiferin exerts a positive effect on alleviating IVDD by inhibiting the inflammatory response of HNPCs, increasing the loss of major components that contributes to the progression of IVDD, and preserving mitochondrial function by reducing ROS production, which reduces the expression of apoptosis-related proteins. In our future studies, we expect to develop mangiferin as a clinical treatment for IVDD.

## Figures and Tables

**Table 1:** Primers for real-time PCR.

Source	Target	Forward primer, 5′-3′	Reverse primer, 5′-3′
Human	COX-2	GGAACTTTCTGGTCCCTTCAG	TGTGTTTGGAGTGGGTTTCA
	iNOS	GCCAAGCTGAAATTGAATGAGGA	TTCTGTGCCGGCAGCTTTAAC
	MMP-13	TGCTGCATTCTCCTTCAGGA	ATGCATCCAGGGGTCCTGGC
	ADAMTS-5	GAACATCGACCAACTCTACTCCG	CAATGCCCACCGAACCATCT
	Collagen-II	TGGACGATCAGGCGAAACC	GCTGCGGATGCTCTCAATC
	Aggrecan	ACTCTGGGTTTTCGTGACTCT	ACACTCAGCGAGTTGTCATGG
	Bcl-2	AACATCGCCCTGTGGATGAC	AGAGTCTTCAGAGACAGCCAGGAG
	Bax	CCCGAGAGGTCTTTTTCCGAG	CCAGCCCATGATGGTTCTGAT
	c-caspase-3	GACTCTGGAATATCCCTGGACAACA	AGGTTTGCTGCATCGACATCTG
	NF-*κ*B_1_	TATTTGAAACACTGGAAGCACG	CCGGAAGAAAAGCTGTAAACAT

## Data Availability

The data collected for the study are available from the corresponding author upon request.

## Abbreviations

IVDD: Intervertebral disc degeneration
HNPCs: Human nucleus pulposus cells
LBP: Low back pain
IVD: Intervertebral disc
ROS: Reactive oxygen species
NP: Nucleus pulposus
Col-2: Type II collagen
ECM: Extracellular matrix
ADAMTSs: A disintegrin and metalloproteinase with thrombospondin motifs
c-caspase-3: Cleaved-caspase-3
TFAM: Mitochondrial transcription factor A
OPA1: Optic Atrophy 1
Drp1: Dynamin-related protein 1
pI*κ*B*α*: Phosphorylated I*κ*B*α*
pp65: Phosphorylated p65
MRI: Magnetic resonance imaging
IHC: Immunohistochemical
HE: Hematoxylin and eosin
IF: Immunofluorescence
TEM: Transmission electron microscopy.
